# Simultaneous targeting of Eph receptors in glioblastoma

**DOI:** 10.18632/oncotarget.10978

**Published:** 2016-08-01

**Authors:** Sara Ferluga, Carla Maria Lema Tomé, Denise Mazess Herpai, Ralph D'Agostino, Waldemar Debinski

**Affiliations:** ^1^ Department of Cancer Biology, Radiation Oncology and Neurosurgery, Brain Tumor Center of Excellence, Comprehensive Cancer Center of Wake Forest Baptist Medical Center, Wake Forest School of Medicine, Medical Center Boulevard, Winston-Salem, NC 27157, USA; ^2^ Department of Neurobiology and Anatomy, Wake Forest School of Medicine, Medical Center Boulevard, Winston-Salem, NC 27157, USA; ^3^ Department of Biostatistical Sciences, Section on Biostatistics, Wake Forest University Health Sciences, Winston-Salem, NC, 27157, USA

**Keywords:** Eph receptors, ephrin-A5, glioblastoma, cytotoxin, molecular targeting

## Abstract

Eph tyrosine kinase receptors are frequently overexpressed and functional in many cancers, and they are attractive candidates for targeted therapy. Here, we analyzed the expression of Eph receptor A3, one of the most up-regulated factors in glioblastoma cells cultured under tumorsphere-forming conditions, together with EphA2 and EphB2 receptors. EphA3 was overexpressed in up to 60% of glioblastoma tumors tested, but not in normal brain. EphA3 was localized in scattered areas of the tumor, the invasive ring, and niches near tumor vessels. EphA3 co-localized with macrophage/leukocyte markers, suggesting EphA3 expression on tumor-infiltrating cells of bone marrow origin. We took advantage of the fact that ephrinA5 (eA5) is a ligand that binds EphA3, EphA2 and EphB2 receptors, and used it to construct a novel targeted anti-glioblastoma cytotoxin. The eA5-based cytotoxin potently and specifically killed glioblastoma cells with an IC_50_ of at least 10^−11^ M. This and similar cytotoxins will simultaneously target different compartments of glioblastoma tumors while mitigating tumor heterogeneity.

## INTRODUCTION

Glioblastoma (GBM) is the most common and most aggressive type of primary brain tumor; it has a dismal prognosis in adults [1] and despite extensive efforts, median survival for patients with GBM remains below 15 months [2, 3]. Limiting factors in GBM treatment include tumor cell resistance to chemotherapy and radiation therapy, and apoptosis in general [4]. Newly identified glioma stem-like cells (GSCs) are suspected to exhibit particular resistance to chemotherapy and radiation therapy [5, 6]. Brain tumors are also more difficult for drugs to reach because of the blood-brain tumor barrier (BBTB) and blood-brain barrier (BBB) [7]. Convection-enhanced delivery (CED) is one of the potentially most effective strategies to overcome these barriers by delivering the drug directly to tumor or around the tumor resection cavity [8, 9].

Molecular-targeted therapy aims at improving specific drug delivery to tumor cells and minimizing damage to healthy tissue [10]. Two promising pharmaceutically targetable biomarkers for GBM include interleukin-13 receptor α chain variant 2 (IL-13RA2) [11] and the Eph receptor A2 [12–15]; together, these are overexpressed in over 90% of GBMs [16]. Recently, the Eph receptor A3 has been recognized as a promising target in GBM. The role of EphA3 is to maintain the de-differentiated state of tumor cells in the more aggressive mesenchymal subtype [17, 18]. EphA3 depletion or treatment with specific anti-EphA3 monoclonal antibody reduced cell tumorigenicity *in vivo* by targeting tumor-initiating cells [17].

Both Eph receptors A3 and A2 belong to the EphA subfamily of receptor tyrosine kinases (RTKs) [19]. Eph receptors are the largest sub-family of RTKs, with 16 known members. They are divided into “A” and “B” sub-classes, and nearly all are activated by ephrin (*Eph* family *r*eceptor *in*teracting proteins) ligands that belong to the corresponding class [20, 21]. These receptors are activated upon binding with their cognate ephrin ligands, which induces receptor clustering followed by internalization and degradation (Supplementary Table S1) [20, 21]. Eph receptors and their corresponding ligands play critical functions during early embryogenesis and development [22], and in various pathologies, malignancies, and injuries in adults [23]. Eph receptors A3 and A2 have been related to several malignancies, including androgen-independent prostate tumor [24], hepatocellular carcinoma [25], hematological cancers, and GBM [12, 15, 26]. We have previously documented that EphA2 as a targetable receptor in GBM [15, 27]. Of interest, a receptor from the B family of Ephs, EphB2, is overexpressed in GBM and correlates with reduced cellular proliferation and increased migration *in vitro* in a xenograft model of GBM [28, 29]. EphB2 was also suggested as a prognostic factor in human pancreatic cancer [30]. Hence, the Eph family of receptors plays an important role in oncological disorders.

Bacterial- and plant-based cytotoxins and immunotoxins have been employed in molecular-targeted therapies to specifically attack malignant cells overexpressing cell surface receptors [31–33]. Targeted toxins have already been used in GBM treatment, showing high potency *in vitro* and promising results *in vivo* when administered locally by CED [11, 34]. Bacterial toxins like *Pseudomonas* exotoxin A (PE) or *Diphtheria* toxin enter the cells by receptor-mediated endocytosis and, following the endosomal pathway through the trans-Golgi and the endoplasmic reticulum, they translocate to the cytoplasm. There they block protein synthesis by ADP-ribosylation of elongation factor-2 (EF-2) thereby causing cell death [11, 32, 35].

In the present work, we document over-expression of the EphA3 receptor, in conjunction with over-expression of the EphA2 and EphB2 receptors, in various compartments of GBM tumors. We also exploited ephrin-A5 (eA5) as a targeted ligand in the production of a conjugate cytotoxin, taking advantage of its ability to bind EphA2, EphA3, and EphB2 [36, 37]. Our data demonstrate that we can simultaneously target all three receptors over-expressed in GBM that localize to different tumor compartments, therefore potentially eliminating tumor-initiating and differentiated cells, neovasculature, infiltrating tumor cells and abnormal cells in the tumor microenvironment.

## RESULTS

### EphA3 is upregulated in GBM cells under tumorsphere-forming conditions

**Figure 1:**
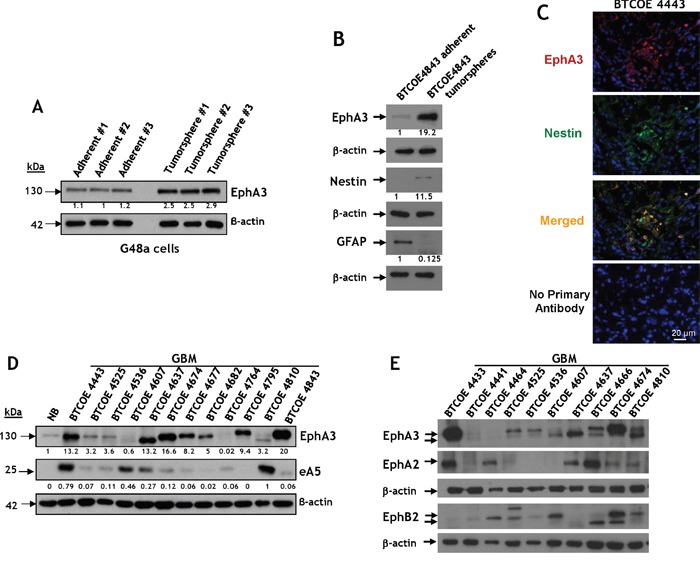
Immunoreactive profile of EphA3 in GBM tumorspheres and snap-frozen tumor specimens **A.** Western blot analysis of EphA3 expression in a G48a GBM cell line grown under standard (left) or under tumorsphere-promoting culture conditions [46, 50]. **B.** Same as in *A*, but the GBM cells were never passaged and derived from the human specimen BTCOE4843. Nestin and GFAP imunoreactivity was also examined. **C.** Immunofluorescent staining of EphA3 (red) and Nestin [50] (green) in a BTCOE4443 human GBM specimen *in situ*. Nuclei are stained with DAPI (blue). **D.** Western blot analysis of EphA3 and eA5 expression in 12 GBM (grade IV astrocytoma) human specimens compared to normal human brain. **E.** Same as in *D*, but the GBM specimens were examined also for the presence of EphA2 and EphB2.

The Eph family of receptors shows promise for pharmaceutical development to find new targetable receptors in GBM [27, 38, 39]. In an effort to find new targetable receptors in GBM, including glioma stem-like cells (GSCs), G48a GBM cells were grown under tumorsphere-forming conditions. Microarray data analysis showed that one of the most significantly up-regulated genes was *EphA3*, among other genes like *Thsd7A, Msi2, Trpm8, and Gpm6A*; the genes most down-regulated were *Tk1, Fst, Anln, AnxA, and Stxbp6*. Protein levels of EphA3 also increased 2-3 fold compared to standard in-adherence growing G48a cells (Figure 1A). This difference in protein expression was much greater (19-fold) in non-passaged GBM cells obtained from the specimen BTCOE4843 (Figure 1B). In addition, we observed co-staining of EphA3 with one of the GSC marker Nestin in a GBM specimen *in situ* (Figure 1C). These results are consistent with a potential role for EphA3 in tumor-initiating cell populations and its largest presence in the mesenchymal subtype of GBM, which has been recently reported by others [17].

### EphA3 is overexpressed in GBM specimens, but not normal brain

We next analyzed specimens of primary brain tumors for the presence of EphA3. The receptor was over-expressed in 7 of 12 of GBM tumor lysates (58%) but not in normal brain (Figure 1D). Only two specimens of this group of GBMs contained EphA3, at very low levels (BTCOE4607 and 4764). The presence of eA5 was variable and the ligand was over-expressed in 33% of tumors (Figure 1D). We screened more normal brain samples and found no to negligible expression of the receptor (Supplementary Figure S1A); only the specimen obtained from a trauma victim had more readily detected EphA3 (Supplementary Figure S1A, NB4656).

For comparative purposes and for assessing heterogeneity of the receptors over-expression, we analyzed GBM specimens for EphA3, EphA2 and EphB2 receptors in the same tumors. The results for individual receptors were similar to the previously reported by us and others [15, 17, 28, 29]. Only one specimen of GBM, BTCOE4441, did not express appreciably any of the three receptors (Figure 1E). EphA3 was also present in all 4 anaplastic oligodendrogliomas examined (WHO grade II/III) and less so in meningiomas and lower grade astrocytomas (WHO grade II) (Supplementary Figure S1B). Therefore, targeting the three Eph receptors would cover vast majority of patients with GBM.

**Figure 2:**
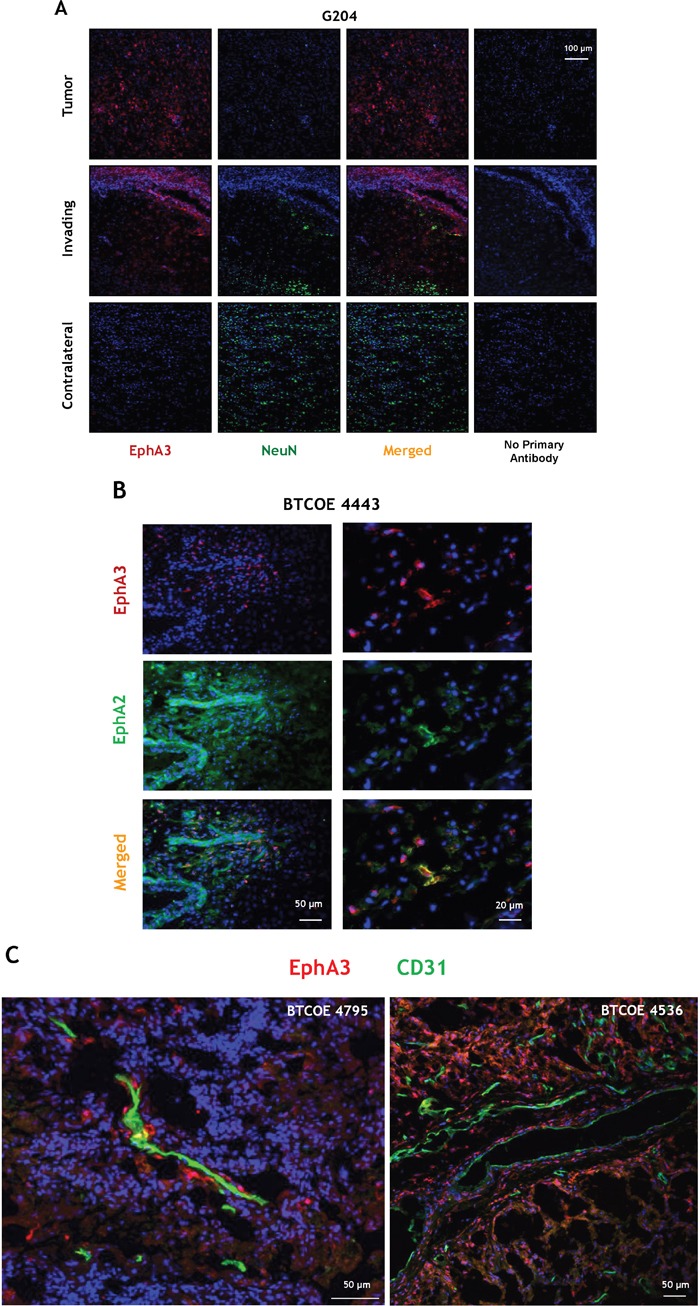
Immunofluorescent staining of EphA3 in GBM **A.** Immunofluorescent staining of EphA3 (red) and NeuN (green) within the tumor, invading and contralateral areas in a patient who died from GBM (specimen G204). **B.** Immunofluorescent staining of EphA3 (red) and EphA2 (green) in a BTCOE4443 human GBM specimen. **C.** Confocal immunofluorescent staining of EphA3 (red) and CD31 (green) in two GBM specimens using stacked 2D images. Nuclei are stained with DAPI (blue).

**Figure 3:**
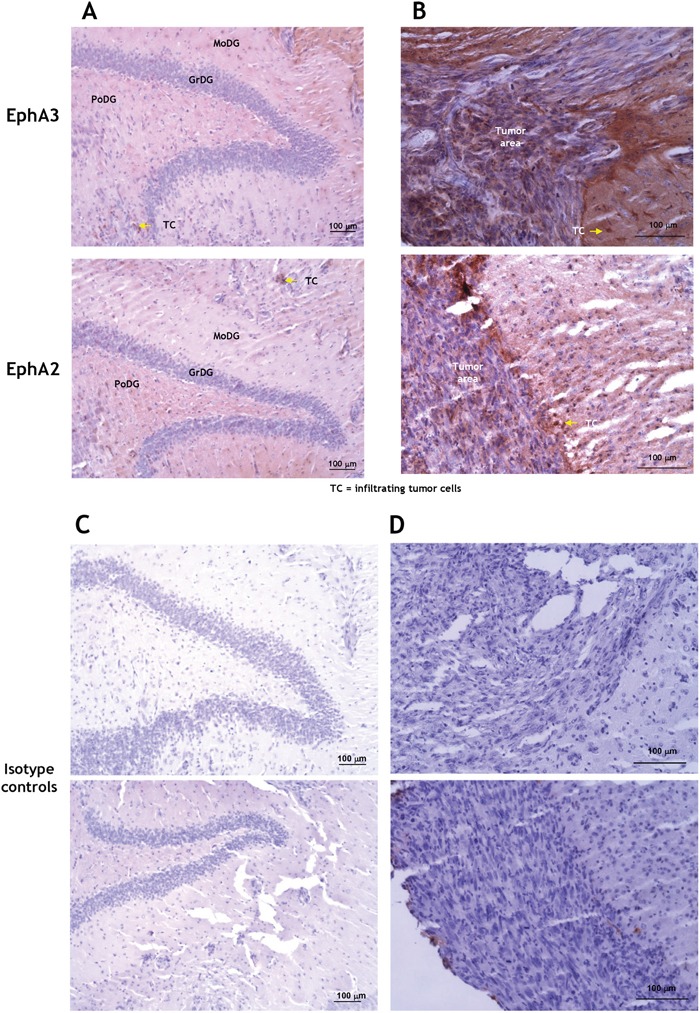
Immunohistochemistry for EphA3 and EphA2 in a mouse brain carrying G48a tumors The hippocampal area **A.** and tumor-normal brain margin **B.** were examined. The hippocampal area did not stain specifically for either the receptor, except for tumor-infiltrating cells (arrows). **C.** and **D.** are the respective isotype-matched controls. MoDG, molecular layer dentate gyrus; GrDG, granular layer dentate gyrus; PoDG, polymorphic layer dentate gyrus; TC, infiltrating tumor cells.

The presence of EphA3 was also examined by immunofluorescent staining of a brain of patient with GBM (G204; donated brain for research). EphA3 was largely present in scattered areas within the tumor and of the invading ring, but not in the contralateral side (Figure 2A). To detect EphA3 on neuronal cells, we co-stained the sections with NeuN antibody. We observed no co-staining of EphA3 with NeuN (Figure 2A). EphA3 and EphA2 localization was also analyzed in another human GBM specimen (Figure 2B). EphA3 was detected in the perivascular space, but it showed a limited co-staining with EphA2 within the tumor area (Figure 2B). EphA2 was found on endothelial cells of tumor neovasculature, as reported [15], and in the surrounding areas (Figure 2B). This was further emphasized by staining GBM specimens for EphA3 and CD31 (Figure 2C and Video 2C, and Figure 2SA-2SB). Moreover, EphA3 or EphA2 receptor could not be detected specifically in the hippocampal region of mouse brain (Figure 3A), in which an EphA3/EphA2-positive tumor was grown (Figure 3B); Figure 3C-3D represent respective controls. Thus, EphA3 is not detected in the areas of neuronal regeneration in the brain.

### EphA3 and EphA2 are highly expressed in GBM cells

**Figure 4:**
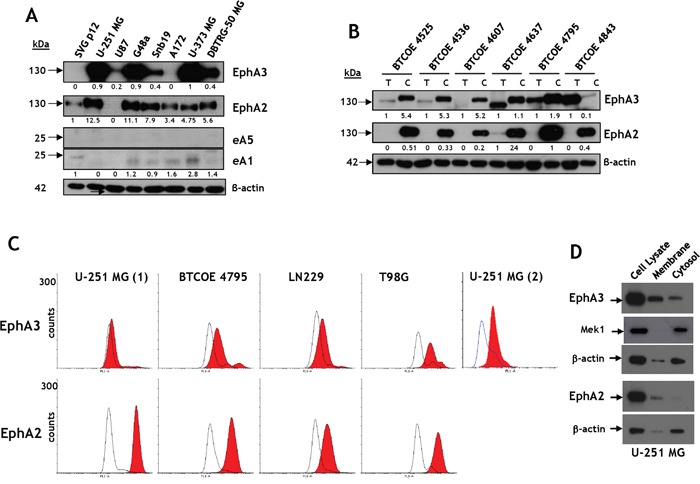
Eph receptor A3 is over-expressed in GBM cell lines and tumors **A.** Western blot analyses of EphA3 and EphA2 and their ligands, eA5 and eA1, respectively, in brain tumor cell lines and in SVG p12 glial cells. **B.** Western blot analyses of EphA3 and EphA2 in GBM primary cells compared to tumor specimens they were derived from. **C.** Flow cytometry for EphA3 and EphA2 in established GBM cell lines. U-251 MG (1) was performed using full antibody while U-251 MG (2) was performed using in-house made scFv fragment of anti-EphA3 antibody. **D.** The presence of immunoreactive EphA3 and EphA2 in membrane and cytosol/cytoskeleton fractions of U-251 MG cells.

EphA3, EphA2, and eA5 and eA1 protein levels were studied by Western blot in several established human GBM cell lines. The receptors displayed similar, but not identical immunoreactive profiles, and were highly overexpressed in most cell lines tested compared to transformed SVGp12 glial cells (Figure 4A). Interestingly, the Eph receptors ligands eA5 and eA1 were absent or detected at much lower levels than in tissue specimens (Figure 4A).

EphA3 and EphA2 protein levels were also evaluated in low-passage GBM cell explant lines and compared to those in tumors they were derived from. The EphA3 receptor was over-expressed in around half of tumor specimens and in most of the isolated cell lines (Figure 4B). However, in a highly cystic GBM sample (BTCOE4843), EphA3 was present in a tumor lysate, but barely detectable in tumor-derived cells. These results suggested EphA3 over-expression in a population of cells in the tumor microenvironment that are not necessarily transformed tumor cells. The tumors used in this particular experiment had lower levels of the EphA2 receptor initially, but the cells cultured from these tumors exhibited high expression of the receptor (Figure 4B). In this assay and others, immunoreactive bands corresponded to EphA3 receptors of different mobilities on gels (e.g., Figure 1E and S1B). This suggests that the receptor might have a different post-translational status.

Next we treated lysates of GBM specimens (BTCOE4536 and 4637) with PNGaseF (Supplementary Figure S3A) and EndoH (Supplementary Figure S3B). Both enzymes caused a release of smaller molecular weight forms of immunoreactive EphA3, confirming that the receptor undergoes glycosylation (Supplementary Figure S3) Furthermore, EphA3 and EphA2 were detectable in U-251 MG, BTCOE4795, LN229, and T98G GBM cells by flow cytometry (Figure 4C); better detection of EphA3 was obtained with the use of single-chain Fv (U-251 (2)) than whole antibody (U-251 (1)). We further studied the presence of the receptors in different cellular compartments by separating membrane from cytosolic fraction of GBM cells. Both EphA3 and EphA2 were associated with membrane and cytosolic/cytoskeleton fractions (Figure 4D).

The results suggest that the expression of EphA3, EphA2, eA1, and eA5 in cells in culture differ from their expression in tumor specimens pointing to an elevated and more frequent expression of the receptors in cells than tumors and the opposite true for the ligands.

### EphA3 co-stains with macrophage/leukocyte markers

**Figure 5:**
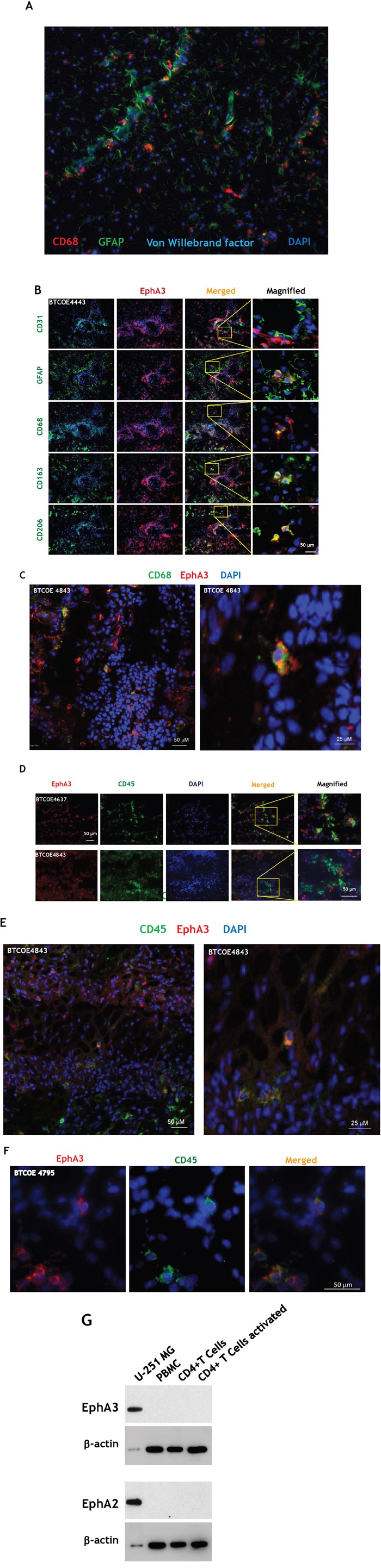
EphA3 co-stains with macrophage/leukocyte markers **A.** Immunofluorescent staining of CD68, GFAP and Von Willebrand factor in a GBM specimen. **B.** Immunofluorescent staining of EphA3 (red) and CD31, GFAP, CD68, CD163, and CD206 on consecutive frozen sections of BTCOE4443 human GBM specimen. Nuclei are stained with DAPI (blue). Selected areas were magnified (last column on the right). **C.** Confocal immunofluorescent staining of EphA3 and CD68 in a GBM specimen using stacked 2D images. **D, E.**-stacked 2D images, and **F.** Confocal microscopy of co-staining of EphA3 and CD45 in GBM specimens. **G.** CD4+ T cells do not express the EphA3 receptor. Western blot analysis of peripheral blood mononuclear cells (PBMC), CD4+ and CD4+ activated T cells were probed for EphA3 immunoreactivity.

Macrophages often localize along blood vessels, in the perivascular space in GBM (Figure 5A). Hence we further analyzed the expression and localization of EphA3 on consecutive sections of the GBM specimens BTCOE4443, BTCOE4843 and BTCOE4795. EphA3 also showed a modest co-staining with glial fibrillary acidic protein (GFAP) (Figure 5B and Supplementary Figure S4A). We next examined EphA3 in relation to macrophages because these cells highly infiltrate gliomas, contributing to total tumor mass [40]. We stained the specimens for three markers of cells of monocyte/macrophage lineages: CD68, CD163, and CD206. Surprisingly, all three monocyte/macrophage markers co-stained with EphA3 in a sub-population of cells surrounding tumor vasculature and in the core of the tumor (Figure 5B and Supplementary Figure S4A). To further study the possibility of co-localization of EphA3 receptor and bone marrow-derived cells, we performed confocal microscopy. We found that CD68 and EphA3 co-stain in a GBM tumor microenvironment indeed (Figure 5B and Supplementary Figure S4A; Figure 5C and Video 5C, and Supplementary Figure S4B-S4C). In addition, we stained for a leukocyte marker CD45 and EphA3 by immunofluorescence (Figure 5D) and performed Z-stack confocal microscopy (Figure 5E-5F and Video 5E, and Supplementary Figure S4D. Similarly to macrophage markers, CD45 co-stained with EphA3 in a subgroup of cells. In further analysis of immune cells, we did not find any expression of the EphA3 receptor in the activated CD4+ T cells (Figure 5G).

### EA5 conjugate to pseudomonas exotoxin A (eA5-PE-C) potently kills GBM tumor cells

**Figure 6:**
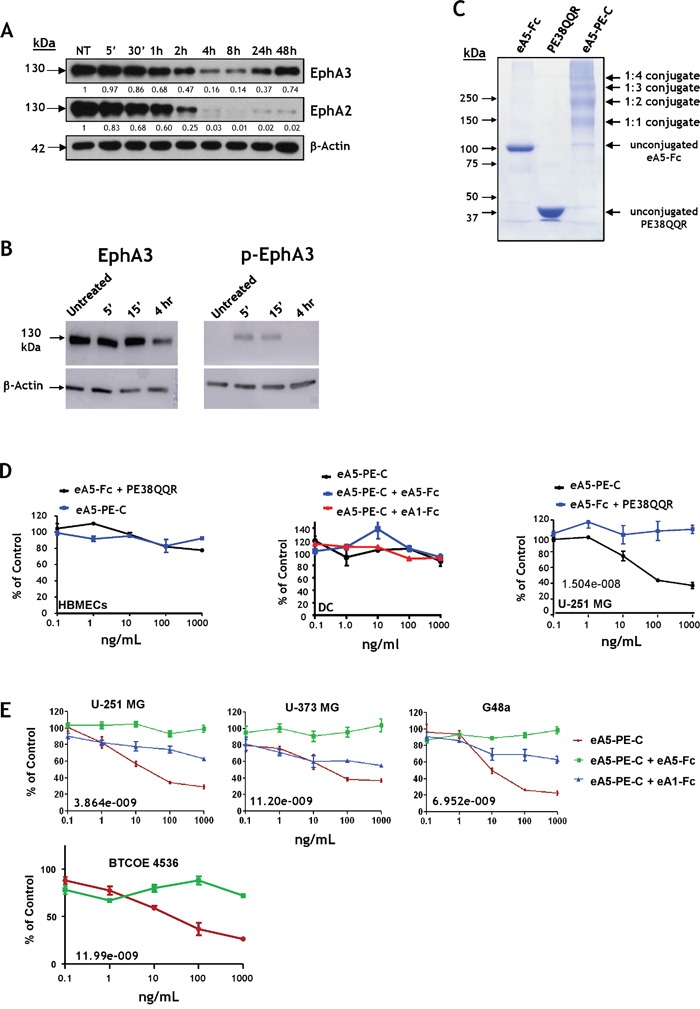
EA5-Fc based anti-GBM cytotoxin **A.** Western blot analysis of EphA3 and EphA2 degradation following treatment with 1 μg/mL of eA5-Fc in U-251 MG cells. **B.** eA5-Fc (made in-house) causes down-regulation (left panel) and phosphorylation at Y779 (right panel) of the EphA3 receptor. **C.** SDS-PAGE of eA5-Fc, PE38QQR and the derived eA5-PE-C chemically conjugated cytotoxin. **D.** MTS/PMS cell viability assay on normal brain endothelial (HBMEC) and dendritic cells (DC), and U-251 MG GBM cells treated with eA5-PE-C cytotoxin or a mixture of eA5-Fc+ PE38QQR for 48 hrs. **E.** MTS/PMS cell viability assay in U-251 MG, U-373 MG, G48a and low passage BTCOE4536 GBM cells using eA5-PE-C and blocking with unconjugated eA1 and eA5 ligands. IC50s (ng/ml) are indicated in the individual panels. To analyze data, we used a 2-way analysis of variance (ANOVA) model with dose (5 levels) and group (3 levels) as factors (see methods).

Having established that EphA3 and EphA2 together are promising molecular targets in GBM tumor cells, tumor neovasculature (EphA2) [15], tumor-initiating [12, 17] and tumor-infiltrating cells of monocytic origin, we focused on combinatorial targeting of these receptors. The EphA3, EphA2, and EphB2 receptors are recognized by eA5 [36] (Supplementary Table S1). We produced a recombinant dimeric form of eA5 in fusion with the Fc region of human IgG_1_ (eA5-Fc) (Supplementary Figure S5A). The recombinant homodimer (created in our laboratory) showed the same mobility pattern as the products obtained from commercial sources like eA5-Fc and eB2-Fc on SDS-PAGE (Supplementary Figure S5B). The commercial eA5-Fc may undergo some proteolytic degradation, unlike our constructs in which eA5 no longer has a proteolysis-sensitive site (Supplementary Figure S5B-S5C) [41]. EA5-Fc was active in inducing EphA2 receptor degradation in U-251 MG cells during 4 hours of treatment, a phenomenon characteristic of the internalized Eph receptors [27] (Figure 6A). The dimeric ligand induced EphA3 degradation from 2 to 24 hours post-treatment; protein levels were completely restored at 48 hours compared to untreated cells. EphA2 showed pronounced degradation starting at 4 hours of treatment, and even at 48 hours, protein levels did not recover (Figure 6A). Moreover, in-house made eA5-Fc caused EphA3 to be phosphorylated at Y779 (Figure 6B) Thus, our eA5-Fc exhibits the expected biological activity. Subsequently, we produced a dimeric eA5-PE38QQR cytotoxin as a chemically conjugated form termed eA5-PE-C (Figure 6C). The conjugated cytotoxin derived from eA5-Fc chemically linked to PE38QQR [42] produced several conjugates; most resulted in a 1:1 or a 1:2 stoichiometric ratio between ligands and toxins.

Initially, the two cytotoxins were tested in human brain normal microvascular endothelial cells (HBMEC), human dendritic cells, and U-251 MG tumor cells using MTS/PMS cell viability assays. We did not see any killing effect in normal HBMEC or dendritic cells after treatment with conjugated cytotoxin or a mixture of eA5-Fc and PE38QQR (Figure 6D). Conversely, we observed a potent killing effect of eA5-PE-C in U-251 MG cells (Figure 6D). Assuming a 1:1 stoichiometric linkage between eA5-Fc and PE38QQR, the IC_50_ of the conjugate was in a range of 10^−11^ M. The mixture of eA5-Fc and PE38QQR produced no effect in GBM cells (Figure 6D). Even though the killing curves did not reach values near zero in a colorimetric cell viability assay based on enzymatic activity, in live/dead tests the vast majority of cells appeared to be dead at 10 ng/ml of conjugate, and almost all cells were dead at 1,000 ng/ml (Supplementary Figure S6).

We further analyzed the effect of eA5-PE-C conjugate in U-251 MG, U-373 MG, G48a and low passage BTCOE 4536 GBM cells (Figure 6E). As expected, the cytotoxin was very active in killing these GBM cells (Figure 6E). To confirm the specificity of the cytotoxin in targeting respective receptors, the GBM cells were pre-treated with either eA5-Fc or eA1-Fc. The treatment with eA5-Fc should block both EphA receptors, plus the EphB2 receptor [34]. EA1-Fc binds only EphA2; hence, EphA3 and EphB2 should remain available to the cytotoxin. As expected, the cytotoxin was significantly less active in the three cell lines tested when pre-treated with eA1-Fc, but lost activity when cells were pre-treated with eA5-Fc (Figure 6E). For the U-251 MG data, the group-by-dose interaction was highly statistically significant (p<0.0001), suggesting that the differences between groups were dose-dependent. In addition, due to the highly consistent outcomes across replicates within each group at each dose, the 3 groups were statistically significantly different from each other at each dose (p-values from <0.001 – 0.0039) with the ea5-Fc having highest percent values, eA1-Fc having intermediate values, and eA5-PE-C having the lowest values. For the U-373 MG data, the group-by-dose interaction was highly statistically significant (p=0.0014), suggesting that the differences between groups were dose-dependent. The overall test for differences among the 3 groups was significant (p<0.001) at the 1, 10, 100, and 1,000 ng/ml dose levels. Similar results were obtained for G48a cells.

## DISCUSSION

In the present study, we provide supportive evidence that the EphA3 receptor is an attractive molecular target for GBM. EphA3 was over-expressed in most GBM specimens tested. It also was present in tumor cells, including the invading ring of the tumor, but not in normal brain, including the areas known to contain progenitor cells of the central nervous system. In addition, EphA3 is present in GBM tumor-initiating cells, with a prominent effect on their biological behavior, as demonstrated by others [17]. Here, we also demonstrate that the EphA3 receptor is expressed in GBM tumor-infiltrating cells of bone marrow origin, macrophage/leukocyte cells, which have been implicated in GBM progression [27]. The distribution of EphA3 and EphA2, a receptor that we had previously found in GBM [15], differs. Therefore, it would be advantageous to target these receptors together in a combinatorial approach. The eA5 ligand can potentially fulfill such a role; thus, we generated an eA5-based chimeric cytotoxin, linking the eA5-Fc dimeric ligand to a truncated form of PE [42]. The cytotoxin was effective in targeting GBM tumor cells, triggering potent tumor cell killing.

The Eph family of receptors is amenable to the development of targeted therapies [27, 38]. GSCs are a small population of slow-dividing and self-renewing glioma cells characterized by an increased resistance to chemotherapy and radiotherapy [5]. Because of their role in sustaining tumor growth, it is of great interest to find new molecular markers that will specifically target GSCs. The Eph receptor A3 was upregulated in tumorspheres of established G48a GBM cells and also in never-passaged GBM explant cells. Not only were EphA3 protein levels increased in tumorspheres, we also observed a high degree of co-staining with the cancer stem cell marker Nestin *in situ*. These data together support an important role of EphA3 in tumor-initiating cells, as previously proposed [17]. We have previously shown that a soluble form of eA1 ligand is still active in inducing EphA2 down-regulation and thus in reducing the oncogenic potential of GBM tumor cells [43]. Similarly, GBM cells overexpressing EphA3 and treated with a monoclonal antibody to deplete the receptor showed markedly reduced tumorigenicity [17].

The tumor microenvironment is a complex mixture of cells that surround and support tumor cells; this mixture can influence tumor progression and therapeutic response/resistance of the treated lesion [26, 44]. Macrophages highly infiltrate gliomas, favoring glioma growth [40]. Interestingly EphA3 was identified as one of the RTK-specific transcripts in bone marrow mesenchymal stromal cells (BMMSCs) [45] and the receptor is overexpressed primarily in the mesenchymal GBM genomic subtype [17]. Taken together, these results suggested a possible role of EphA3 in tumor-infiltrating macrophages/leukocytes. We found that EphA3 co-stains with cells markers of the macrophage/leukocyte lineages CD68, CD163, CD206, and CD45. In addition, recent findings localized EphA3 predominantly to the stromal tumor microenvironment of lung, prostate, and colon cancers, and mouse tumor xenografts [46].

EphA3 is thus present on tumor cells, tumor-initiating cells, infiltrating tumor cells, and tumor-infiltrating cells of monocytic origin. EphA2 is overexpressed in tumor cells, tumor-initiating cells, and in tumor neovasculature. Eph receptors A2 and A3, as well as EphB2, share the property of being activated upon binding with the same ephrin ligand, eA5 [36, 51]. Of importance, eA5 is expressed in gliomas and has a strong tumor suppressing activity in gliomas [52]. We exploited these features to design a novel eA5-based cytotoxin to target GBM cells over-expressing the three receptors. The chimeric cytotoxin was generated by linking eA5-Fc to PE38QQR. The novel eA5-based cytotoxin was neutral to normal cells tested, but effectively killed tumor cells demonstrating overexpression of EphA3, EphA2, and EphB2 receptors.

In summary, similarly to another group, we found EphA3 to be an attractive target in GBM. We produced a highly potent eA5-based cytotoxin that kills tumor cells over-expressing receptors EphA3, EphA2, and also EphB2. These receptors together localize to subpopulations of cells related to tumor progression, invasion, recurrence, and resistance to therapies. EphA2 was previously found in infiltrative [15] and tumor-initiating cells [12] and EphA3 is also overexpressed in the invading ring of the tumor. The possibility of specific killing of residual tumor cells by delivering the cytotoxin locally and safely is of great interest as a new therapeutic option in GBM treatment. Moreover, the eA5-based cytotoxin simultaneously targets several Eph receptors at a time that are overexpressed in various compartments of GBM tumors, but not normal brain. This should allow more comprehensive molecular targeting in this disease, because GBM tumor heterogeneity will be addressed at two levels: 1) different tumor compartments, and 2) differing levels of targeted receptors' expression among different tumor compartments. In addition, the cells evading killing by the eA5-based cytotoxin due to insufficient amount of the targeted receptor expression will be bound by a tumor suppressor, which represents an exceptionally attractive therapeutic scenario. In translation to the clinic, it is envisioned that the cytotoxin will be tested pre-clinically in a canine model of glioma [53] and then administered first to patients with recurrent GBM using advanced reflux-preventing catheters [8] with real-time monitoring of drug's distribution [54].

## MATERIALS AND METHODS

### Cell lines, tissues and reagents

U-251 MG, U-373 MG A-172 MG, U-87 MG, SNB19, DBTRG-50 MG, LN229, and T98G cell lines were obtained from the American Type Culture Collection (ATCC, Manassas, VA) and grown in their recommended media. HBMEC cells were a kind gift of Dr. L. J. Metheny-Barlow and were cultured in the supplier-recommended media. G48a cells were isolated in our laboratory from a human primary high-grade astrocytoma [47]. All human samples were handled according to Wake Forest IRB-approved protocol (#8427).

Early-passage tumor cells were derived from human GBM tumors obtained from the operating room and processed within 20 minutes of resection. Tumors were minced into small pieces and digested with Collagenase II, Collagenase IV, and DNAse (Sigma) for 30 minutes at 37°C. The cell suspension was layered over a ficoll gradient and centrifuged at 300xg for 35 min. The interface was washed twice with phosphate-buffered saline (PBS) and the cells cultured in RPMI-1640 containing 10% fetal bovine serum (FBS) and 4g/L glucose. U-251 MG, U-87 MG, and A-172 MG cells have been authenticated and BTCOE4525, BTCOE4795, and BTCOE4536 cells were validated to the original tumor by Idexx Radil (Columbia, MO). No information regulated by the US Health Insurance Portability and Accountability Act was included in the study, which qualified for exemption #4 of the National Institutes of Health.

### EphA3 and EphA2 down-regulation assays

EphA2 and EphA3 down-regulation assays were performed as previously described [48].

### Recombinant proteins design, expression and purification

The EA5 gene was synthesized based on the GeneBank database (NCBI) sequence AAH75054.1 and cloned into BamHI-EcoRI sites in the modified Baculovirus transfer vector pAcGP67-B (BD Biosciences, San Diego, CA) [48, 49]. Recombinant truncated eA5 (aa. 21-191) [41] was produced in the dimeric form (*C*-terminal Fc tag) in the Baculovirus expression system (BD Biosciences) as previously described [48, 49]. PE38QQR was produced and purified in our laboratory as previously described [42].

### Chemical conjugation and cell viability assays

Protein conjugation was achieved following a previously reported protocol [35] combining eA5-Fc to PE38QQR in a 1:3 molar ratio. The conjugated cytotoxin was additionally purified by size exclusion chromatography. Cell viability assays were performed using the CellTiter 96® AQueous Non-Radioactive Cell Proliferation Assay (MTS) following the instructions of the manufacturer (Promega, Madison, WI).

### EphA3 scFv-Fc production for flow cytometry

Vκ and Vh sequences derived from humanized EphA3 antibody (clone IIIA4) (patent application US20140120114 A1) were synthesized with a glycine-serine linker and inserted in-frame with a honeybee secretion signal and a human IgG_1_ Fc cloned in our laboratory. This construct was transfected into Sf9 cells and the protein was collected from the media. Following purification on a protein G column, the protein was concentrated and filter sterilize. 2 μg/100 μl was used for flow cytometry followed by detection with anti-human Alexa-488.

### Western blots

Cell lysates were prepared by lysing cells in RIPA buffer with proteases and phosphatases inhibitors (Sigma), and separated by 10% SDS-PAGE. Western blotting was performed as previously described [15]. Primary antibodies from Santa Cruz Biotechnology (Santa Cruz, CA) included: EphA3 (C-19), EphA3 (L-18), ephrin-A5 (RR-7), and ephrinA1 (V-18). Other antibodies used were EphA2 (clone D7) (EMD Millipore Corporation, Billerica, MA) and β-actin (Sigma).

### Immunofluorescent staining

Immunofluorescent staining was performed as described previously [15]. Primary antibodies used include: EphA3 C19 (Santa Cruz), EphA2 clone D7 (Millipore, Billerica, MA), NeuN clone A60 (Millipore), GFAP (Santa Cruz), CD31 (Pierce, Rockford, IL), CD68 clone SPM281 (Novus Biologicals, Littleton, CO), CD163 clone 5C6FAT (Novus Biologicals), Nestin (1:200, Santa Cruz) and CD206 clone 15-2 (Santa Cruz).

### Immunohistochemistry

5×10^5^ G48a GBM cells were injected intracranially into brains of nu/nu mice 2 mm to the right and 3 mm rostral to Bregma at a depth of 1 mm. Tumors were allowed to develop for 6 weeks at which time mice were euthanized. Mouse brains were fixed in 10% formalin and embedded in paraffin. Sections were cut at a thickness of 8 μm. Slides were heated at 65°C, de-paraffinized in xylene, and rehydrated. The staining was performed according to our standard protocol. The experiment was performed under Wake Forest IACUC protocol A-14-223.

### Flow cytometry

Cells were detached with EDTA, washed, and resuspended in 100 μl PBS containing 1% bovine serum albumin (BSA). 2×10^5^ cells were blocked with PBS/1% BSA for 1 hour on ice. 2 μg of EphA3 antibody L-18 (Santa Cruz Biotech) or EphA2 clone B2D6 (Millipore) was added and incubated for 2 hours on ice with occasional mixing. Cells incubated with isotype antibody served as controls. Cells were washed 3 times with PBS/1% BSA by centrifugation and resuspended in 100 μl PBS/BSA. 2 μg anti-mouse or anti-human Alexa 488 secondary antibody was added and cells were incubated on ice for an additional 1 hour with occasional mixing. Cells were washed 3 times with PBS/1% BSA by centrifugation and resuspended in 500 μl PBS/BSA. 500 μl of 10% buffered formalin was added to post-fix cells. After washing with PBS/BSA, the cells were analyzed on an Accuri 6 flow cytometer. Data was analyzed using Flowing Software (Turko, Finland).

### Confocal microscopy

Tissue sections were viewed and 0.5 μm z-stacks were acquired with an Olympus Fluoview 1200 confocal microscope (Cellular Imaging Core, Comprehensive Cancer Center of Wake Forest University). Stacked 2D images were obtained in some cases. Images were processed with OlyVIA V2.8.

### Dendritic cells

Myeloid-derived naïve dendritic cells were purchased from AllCells LLC (Alameda, CA). Cells were grown in RPMI-1640 containing 10% FBS, 20 ng/ml GM-CSF (PeproTech, Rocky Hill, NJ) and 20 ng/ml IL-4 (made in our laboratory). Dendritic cells were matured by adding 20 ng/ml TNFα for 72 hours.

### Isolation and activation of CD4+ T cells

Total CD4+ T Cells were isolated from PBMC (ZenBio, Durham, NC) using the Dynabeads Untouched human CD4+ T-Cell Kit (Invitrogen) according to the manufacturer's instructions. The cells were plates at a density of 4 × 10^4^/ml in RPMI-1640 containing 10% FBS, 0.05 mM 2-mercaptoethanol and 30U/ml IL-2 (Peprotech, Rocky Hill, NJ). Cells were activated with CD3/Cd28 magnetic beads (Invitrogen) for 3 days prior to preparation of lysates.

### Tumorsphere preparation

Freshly dissected GBM tumors were digested with collagenase and DNAse. Cells were separated by layering over a Ficoll gradient and centrifuged at 300 xg for 35 min. The interface was collected and washed twice with PBS. Control cells were plated on standard 6-cm dishes for 14 days in RMPI-1640 containing 10% FBS and adjusted to contain 4 g/L glucose. For tumorspheres, cells were plated on HydroCell 6-cm dishes (Thermo Scientific) in serum-free neurobasal media containing B27, N2, (Life Technologies), 20 ng/ml FGF, and 20 ng/ml EGF (PeproTech) for 14 days. Media in all dishes was refreshed every 2-3 days.

### Membrane and cytosolic protein isolation

Membrane and cytosolic proteins from U-251 MG cells were obtained from 100-cm dishes at 90% confluence with ProteoJET Membrane Protein Extraction Kit (ThermoFisher Scientific) according to the manufacturer's instruction.

### Statistical analysis

To analyze data, we used a 2-way analysis of variance (ANOVA) model with dose (5 levels) and group (3 levels) as factors. We first examined whether there was a dose-by-treatment interaction to determine whether changes in outcome differed across the groups over the dose range. If this interaction was significant, then we fit 1-way ANOVA models at each dose to determine whether the 3 groups differed at a particular dose. If the overall 1-way ANOVA had a significant group effect, then we examined the 3 pairwise comparisons between groups within the ANOVA model using an Bonferroni adjusted p-value of 0.0167 to declare a pairwise comparison statistically significant to adjust for the multiple comparisons made at each dose (p=0.05/3 = 0.0167), at which dose the three groups separated from each other. All analyses were performed using SAS Version 9.3.

## SUPPLEMENTARY DATA FIGURES AND TABLE


