# Supplementary material for: Simultaneous targeting of Eph receptors in glioblastoma
Source: Oncotarget. 2016 Aug 1;7(37):59860–76. doi: 10.18632/oncotarget.10978 (PMC5312354; doi:10.18632/oncotarget.10978)

Simultaneous targeting of Eph receptors in glioblastoma

SUPPLEMENTARY DATA

Deglycosylation

10 µg of each lysate was reduced with DTT followed by incubation with the either PNGaseF or EndoH according to the manufacturer instruction (New England Biolabs, Ipswich, MA). Samples without enzyme served at controls. Samples were analyzed by western blot for expression of EphA3.

Live/dead cell analysis

Cells were gently washed with PBS and 100 µl of PBS was added to each well. 100 µl of dye mixture (LIVE/DEAD Viability/Cytotoxicity Kit for mammalian cells, LifeTechnologies) was added according to the manufacturer. The plate was protected from light and incubated for 45 min. Fluorescence was quantified using an Optima Fluorostar plate reader. Photomicrographs were taken with an Olympus IX70 inverted microscope and a Retiga EXi Fast 1394 camera. Images were processed with Image-Pro plus 5.1 software.

EA5-Fc resistance to proteolysis

A total of 1 µg eA5-Fc (made in-house), eA5-Fc (R&D Systems) or recombinant $hIgG_1$ (R&D Systems) was incubated with 250 µl PBS or U-251 MG cell-conditioned medium, for 3 hrs at 37 °C. Samples were separated on a 15% SDS-Page acrylamide under reducing conditions. Proteins were transferred to PVDF membrane and western blot analysis was performed for eA5. (Abnova, Walnut, CA).

SUPPLEMENTARY REFERENCES

1. Dai D, Huang Q, Nussinov R, Ma B. Promiscuous and specific recognition among ephrins and Eph receptors. Biochim Biophys Acta. 2014; 1844:1729-1740.
2. Pasquale EB. Eph-ephrin promiscuity is now crystal clear. Nat Neurosci. 2004; 7:417-418.
3. Pasquale EB. The Eph family of receptors. Curr Opin Cell Biol. 1997; 9:608-615.
4. Pabbisetty KB, Yue X, Li C, Himanen JP, Zhou R, Nikolov DB, Hu L. Kinetic analysis of the binding of monomeric and dimeric ephrins to Eph receptors: correlation to function in a growth cone collapse assay. Protein Sci. 2007; 16:355-361.
5. Choi S, Jeong J, Kim T, Park S. Characterization of ephrin-A1 and ephrin-A4 as ligands for the EphA8 receptor protein tyrosine kinase. Mol Cells. 1999; 9:440-445.

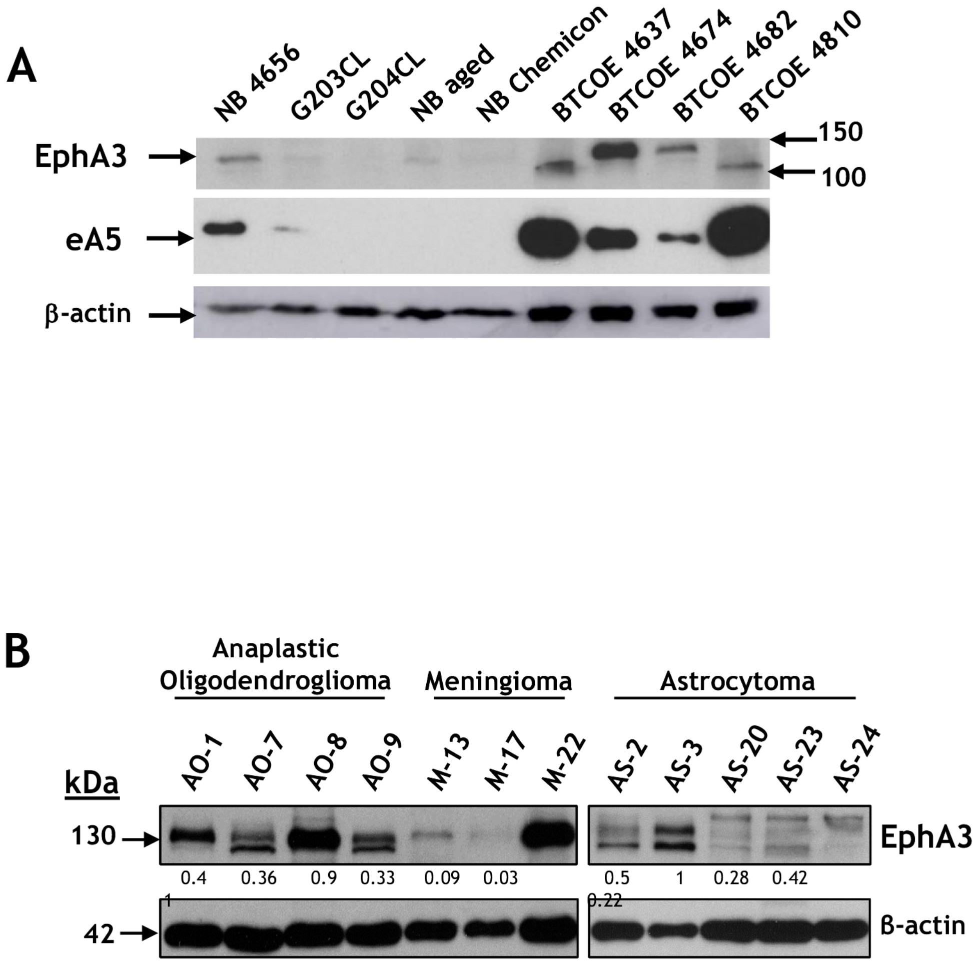

Supplementary Figure S1: Eph receptor A3 is expressed at very low to negligible levels in normal brain lysates compared to GBM tumor lysates. A. Western blot analysis of EphA3 and eA5 expression in 4 GBM human specimens compared to 5 normal human brains. NB 4656, trauma patient; G203CL, G204CL and NB Aged, patient bodies donated for research. **B.** Western blot of EphA3 expression in lower grade gliomas and meningiomas.

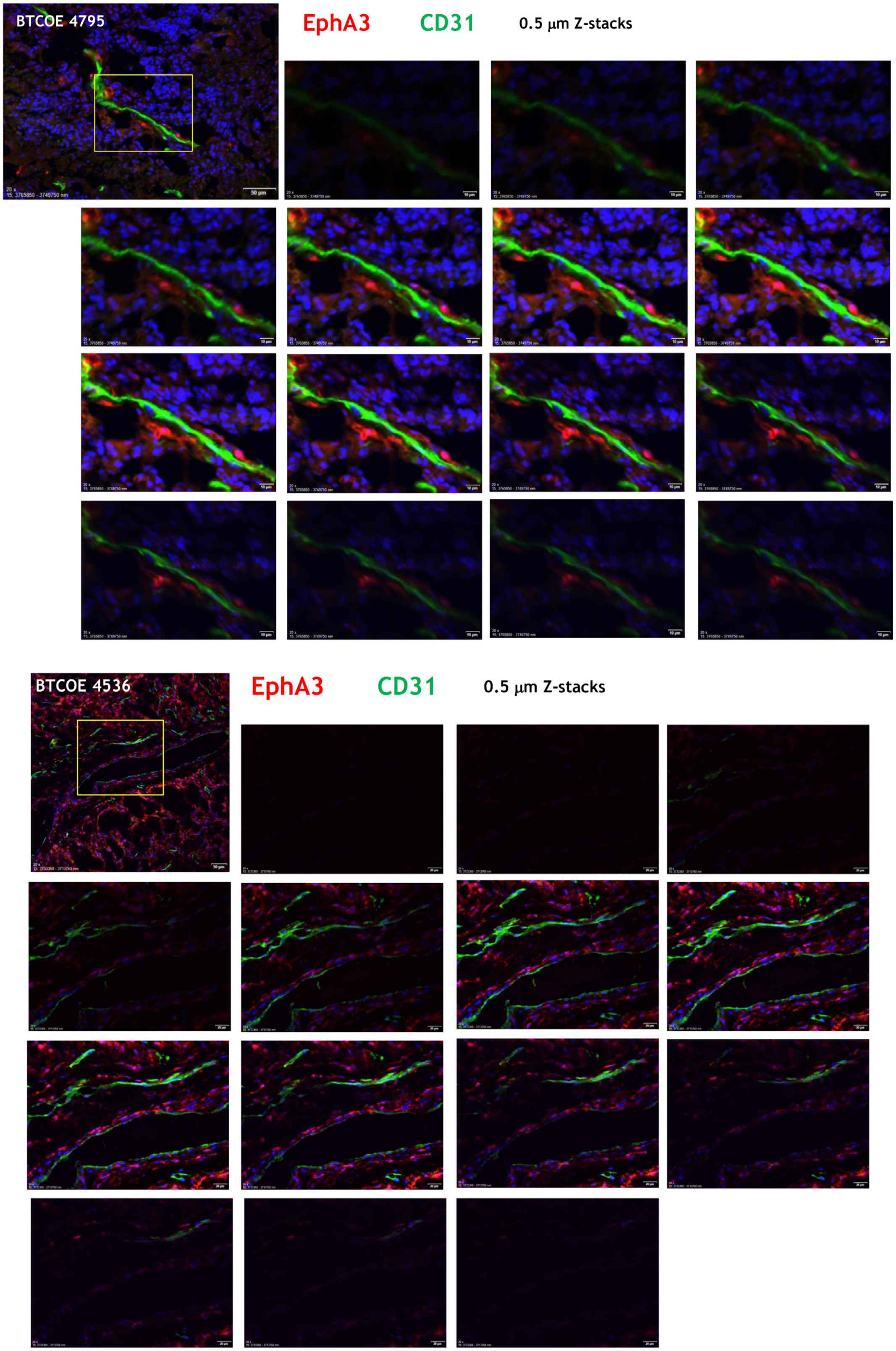

Supplementary Figure S2: Confocal analyses of the EphA3 receptor expression in GBM in the context of CD31 vascular staining. DAPI nuclear staining is in blue.

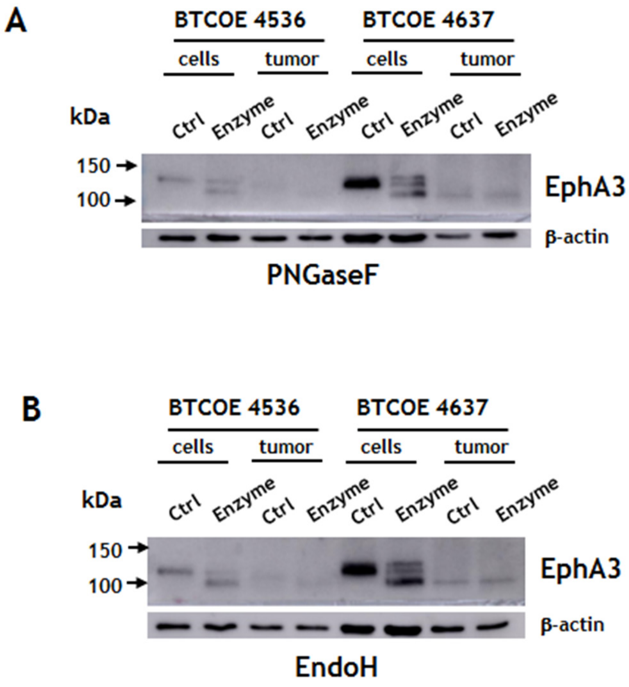

Supplementary Figure S3: EphA3 receptor is glycosylated. Tumor lysates and the tumor-derived cell lysates from four primary tumors were treated with two endoglycosidases, PNGaseF (*top*) and EndoH (*bottom*), both enzymatically remove *N*-linked glycans from glycoproteins.

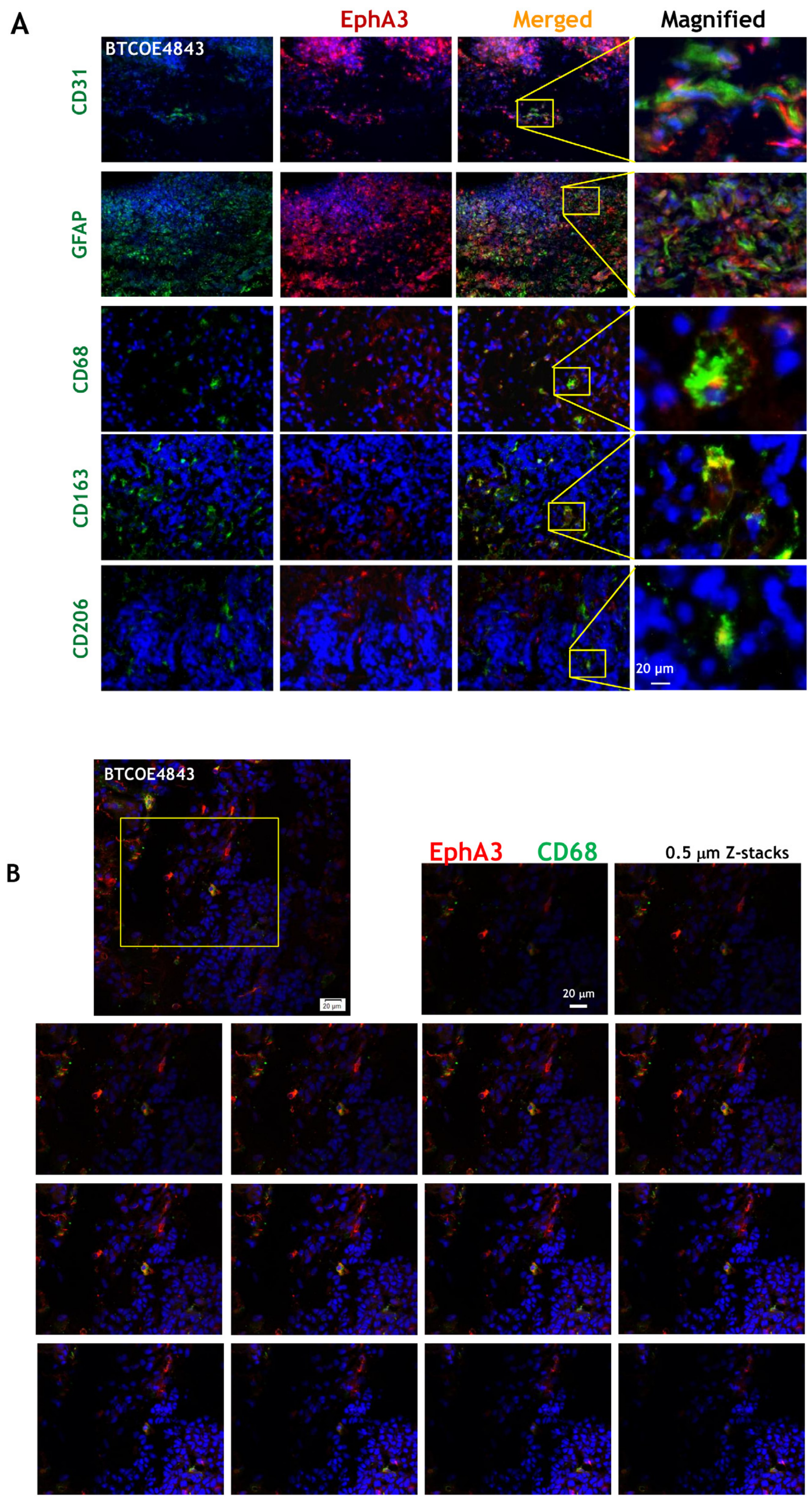

Supplementary Figure S4: EphA3 co-stains with macrophage/leukocyte markers. A. Immunofluorescent staining of EphA3 (red) and CD31, GFAP, CD68, CD163, and CD206 on consecutive frozen sections of BTCOE4843 human GBM specimen. Nuclei are stained with DAPI (blue). Selected areas were magnified (last column on the right).

(*Continued*)

C

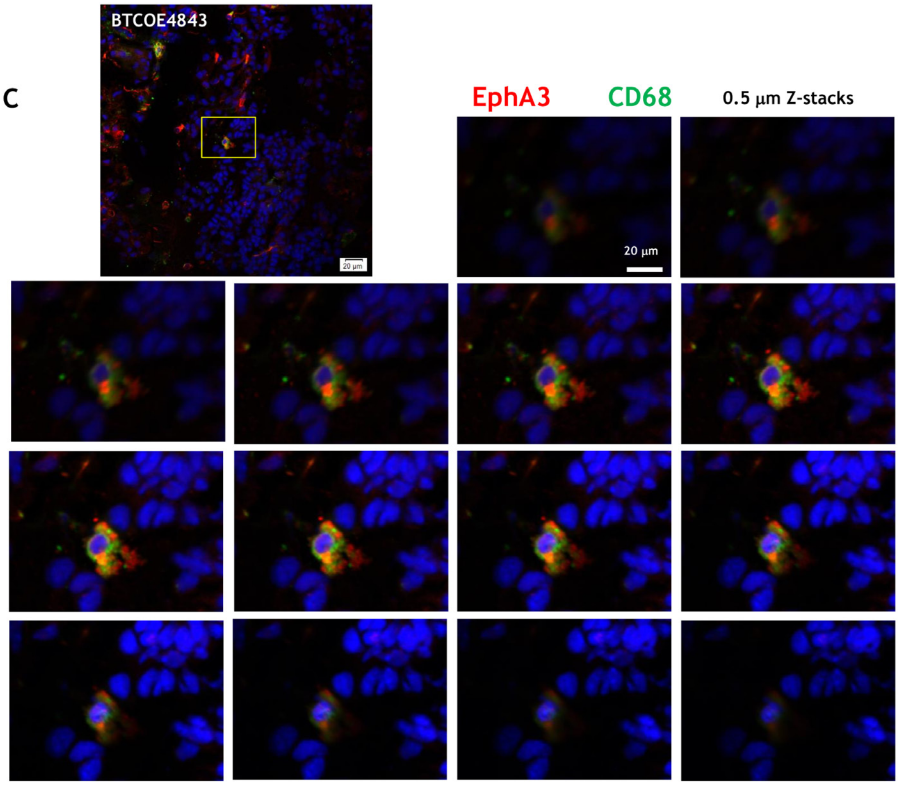

D

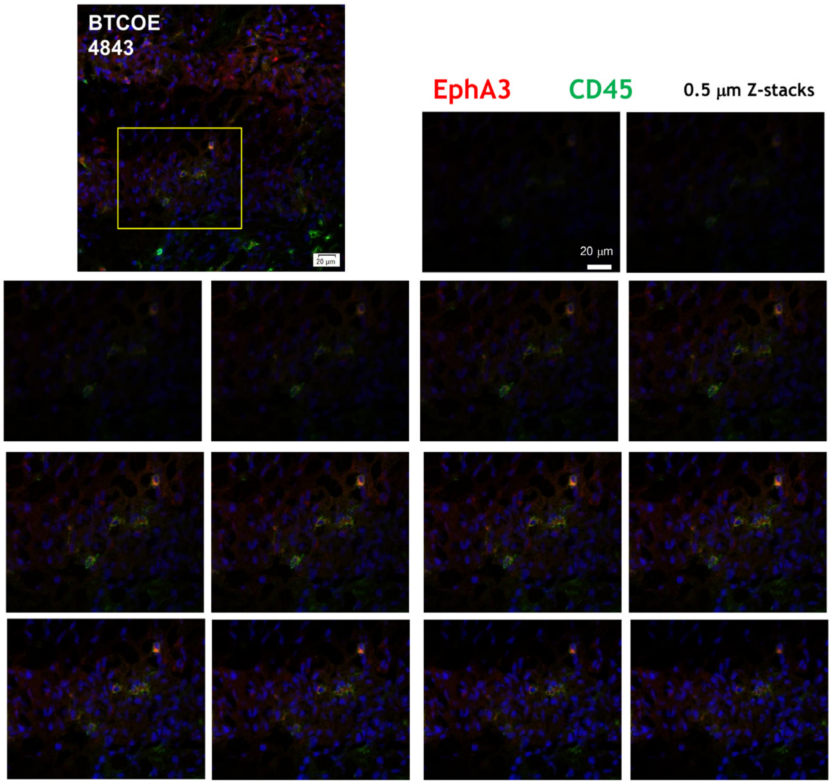

Supplementary Figure S4 (*Continued*): B, C. Confocal immunofluorescent staining of EphA3 and CD68 in a GBM specimen. **D.** Confocal microscopy of co-staining of EphA3 and CD45 in a GBM specimen.

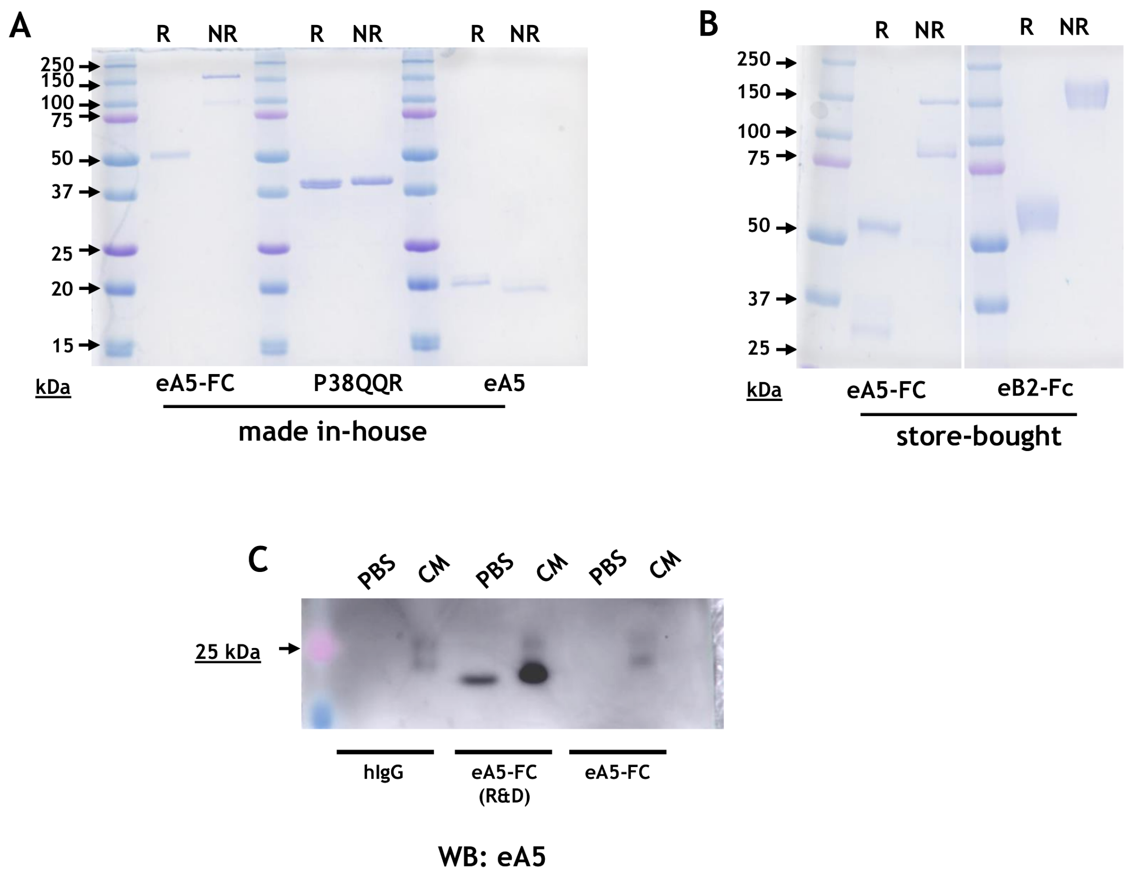

Supplementary Figure S5: The mobility of a dimeric eA5-Fc made in-house is comparable to that of commercially available dimeric eA5-Fc and eB2-Fc on SDS-PAGE. A. Coommassie blue-stained SDS-PAGE of dimeric eA5-Fc, PE38QQR and monomeric eA5 run under reducing (R) or non-reducing (NR) conditions. **B.** Coommassie blue-stained SDS-PAGE analysis of commercially available eA5-Fc and eB2-Fc (R&D Systems) run as in *A* under R or NR conditions. **C.** Proteolytic stability assay. Only the full length, unmodified eA5-Fc (R&D) produced degraded fragments of eA5; see also B.

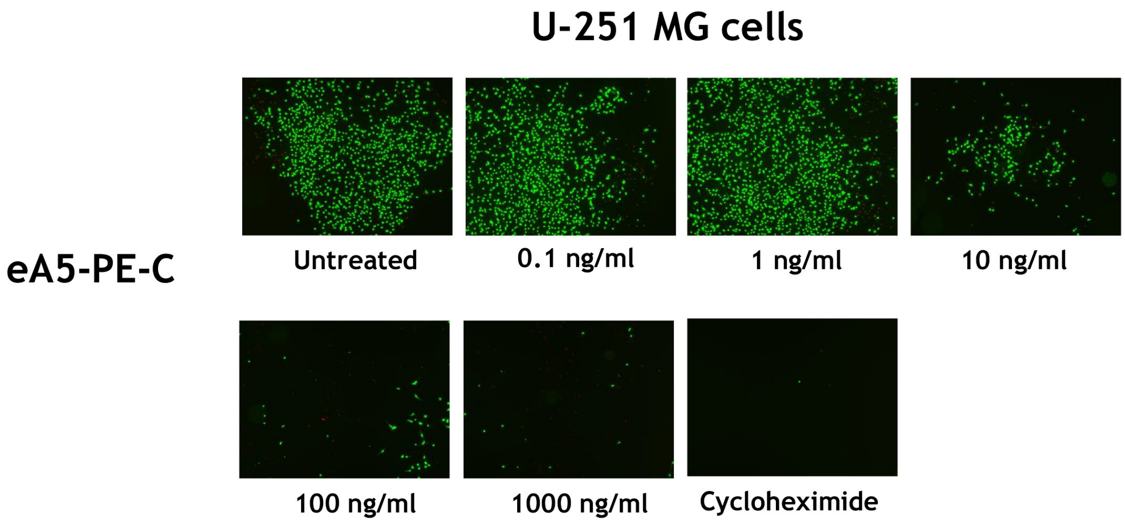

Supplementary Figure S6: EA5-PE-C cytotoxin kills most of the U-251 MG treated cells at a concentration of 10 ng/ml. The Live/Dead assay was performed treating GBM cells with increasing concentration of cytotoxin. Only living cells can be stained. Untreated cells and cycloheximide-treated cells were used and a negative and positive control, respectively.

Supplementary Table S1: Eph receptors and the ephrin ligands binding specificity

Ephs	Ephrins
A1	-A1
A2	-A2, -A3, -A1, -A5, -A4
A3	-A1, -A2, -A5, -A3, -B2
A4	-A4, -A5, -A1, -A2, -A3, -B2, -B3
A5	-A5, -A1, -A2, -A3, -A4
A6	-A2, -A1, -A3, -A4, -A5
A7	-A2, -A3, -A1
A8	-A5, -A3, -A2, -A1, -A4
B1	-B2, -B1, -B3, -A3
B2	-B1, -B2, -B3, -A5
B3	-B1, -B2, -B3
B4	-B2, -B1, -B3

Ligands are listed in an approximate order of decreasing activity and specificity [1-5].

Red: specific and blue: promiscuous.

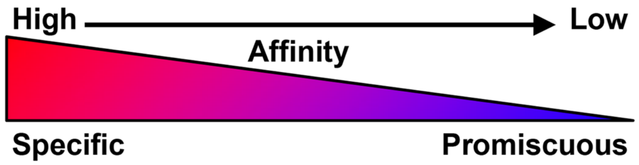

Supplement: Supplementary file 1 [file oncotarget-07-59860-s001.pdf]
